# Understanding delivery of bad news in the emergency department: A scoping review

**DOI:** 10.1371/journal.pone.0358343

**Published:** 2026-09-28

**Authors:** Graham Johnson, Sarah Edwards, Heather Jarman, Elizabeth Peel, Marco Pino

**Affiliations:** 1 School of Social Sciences and Humanities, Loughborough University, Epinal Way, Loughborough, United Kingdom; 2 School of Medicine, University of Nottingham, Queen’s Medical Centre, East Block, Nottingham, United Kingdom; 3 Emergency Department, Royal Derby Hospital, Derby, United Kingdom; 4 Emergency Department Clinical Research Group, St George’s University Hospitals NHS Foundation Trust, London, United Kingdom; European University of Rome: Universita Europea di Roma, ITALY

## Abstract

**Background:**

Breaking bad news is a core component of emergency care, but the emergency department (ED) environment of urgency, crowding, and limited privacy presents challenges for sensitive communication. Many communication frameworks originate from other specialities, but the ED has limited evidence and guidance tailored to its context.

**Objective:**

This scoping review aimed to map the extent, nature, and characteristics of the literature on delivery of bad news in EDs, including terminology, frameworks, stakeholder perspectives, barriers and facilitators, educational resources, reported metrics, and evidence gaps.

**Methods:**

The review followed Joanna Briggs Institute (JBI) methodology and was prospectively registered on the Open Science Framework (OSF https://osf.io/f6q59/overview). Four databases (MEDLINE, EMBASE, EMCARE, CINAHL) and grey literature sources were searched in July 2025. Eligible sources included empirical studies, training evaluations, reviews, guidelines, consensus statements, editorials, commentaries, opinion pieces, educational articles, conference abstracts, and relevant grey literature reporting on adult patients, relatives, or clinicians in ED contexts. Data were charted and synthesised narratively.

**Results:**

From 7985 records, 156 publications were included. Studies originated from 26 countries, though the United States of America (USA) (n = 85) and United Kingdom (UK) (n = 18) dominated the literature. There was a rise in publications from 2010, particularly post–COVID-19. Twenty-five communication frameworks were identified; most were adapted from oncology (with SPIKES most frequently cited). Three frameworks developed specifically for ED practice (GRIEV_ING, 8S, EMTalk) were identified. Stakeholder perspectives were unevenly represented, with a predominance of healthcare professional perspectives. Reported metrics centred on clinician confidence; patient- and family-centred studies were uncommon. Barriers to effective communication consistently included time scarcity, lack of privacy, and insufficient training.

**Conclusions:**

Research on delivering bad news in EDs is dominated by educational and simulation-based studies, and is largely clinician-centred, with limited evidence of patient and family perspectives, with no studies examining long-term outcomes. Reliance on frameworks from other specialties risks overlooking the contextual specifics of emergency care. Future research should prioritise ED-centred frameworks, patient outcomes, and systemic enablers to support compassionate, effective communication in this demanding environment.

## Introduction

Emergency departments (EDs) are characterised as environments defined by high patient acuity, lifesaving, time-critical interventions, and rapid patient turnover. Clinicians have traditionally been trained and resourced to focus on the immediate management of acute medical and surgical emergencies. However, whilst internationally healthcare services differ in how they provide emergency care, most EDs see a broad range of clinical conditions and complexities, ranging across medical, surgical and psychiatric illnesses. Consequently, the differentiated mix of clinical conditions and complexities, managed within a fixed physical environment, contributes to an often over-crowded [1–4] and noisy environment. EDs are commonly the noisiest clinical areas of hospital [5,6] and achieving privacy for sensitive conversations can be difficult.

Unlike settings where clinicians may have longstanding relationships with patients and families, emergency practitioners often meet individuals for the first time, at the moment of crisis, either their own or that of a relative or friend [7,8]. The unpredictability of presentations, rapid evolution of clinical situations, and frequent diagnostic uncertainty compound the challenge of communicating accurately and compassionately.

Staff in EDs frequently report feeling that they lack sufficient time to conduct in-depth discussions due to competing priorities and crowding [1,7,8]. These can be exacerbated during overnight shifts, when senior and specialist staff may be less available, limiting the support and supervision available to less experienced clinicians.

It is perhaps unsurprising therefore that communication is one of the components of ED clinical care most complained about internationally, with studies highlighting that dissatisfaction often stems as much from how information is conveyed, as from the nature of the information itself [9–11]. Inadequate or insensitive delivery of bad news can impact both patient and family experience and has been associated with more intense grief reactions, complicated bereavement, and psychological distress for patients and relatives [12–15]. For clinicians, these interactions can be sources of moral distress, uncertainty, and lead to feelings of inadequacy, particularly when training in communication skills has been insufficient [16,17].

While the clinical imperative to deliver time-critical treatment in this setting has remained unchanged, the responsibility to communicate life-altering news sensitively and effectively has become a more prominent component of the emergency clinician’s role. In recent decades, demographic and epidemiological trends in high and upper-middle income countries have contributed to profound shifts in the nature of emergency care [18]. An ageing population with complex comorbidities, combined with structural pressures on inpatient and outpatient services, has meant that serious and life-limiting diagnoses, such as cancer, transitions to palliative care, and confirmation of the death of a relative have increasingly been first communicated to patients during emergency presentations [14,15]. In some healthcare systems, as many as 50% of cancer diagnoses have been identified in the ED [19,20].

Recent surveys and consensus exercises have underscored the importance of improving communication training and support for emergency clinicians. A national survey in Ireland found that only 39% of less experienced ED clinicians felt comfortable discussing end-of-life care with patients and families, while 79% of all respondents felt that additional training was needed [8]. Similar findings have been documented in Australia, Canada, and the UK, with professional bodies such as the Royal College of Emergency Medicine and the James Lind Alliance identifying end-of-life communication as a research and training priority [21,22]. Canadian emergency medicine residency programs have also recognised palliative care and communication skills as critical curricular gaps [23].

Despite this, the evidence base and training resources produced to guide these conversations have been limited and primarily derived from other specialties such as oncology and palliative care [24–29]. This reliance on evidence and tools developed in non-emergency contexts is potentially problematic, given the distinct characteristics of the ED environment outlined above. When starting this project we were aware of only one structured communication framework, the GRIEV_ING protocol, developed specifically for use in the ED, and it has focused exclusively on delivering news of a death rather than more varied forms of bad news which are more common [30]. Additionally, whilst those working in EDs frequently care for children and young people, communication with these groups present distinct challenges, such as requiring age-appropriate communication with children [31] and supporting parental involvement [32]. Communication in these contexts is supported by a separate body of evidence and guidance and therefore was excluded from this scoping review.

Several other frameworks have been developed to structure the process of breaking bad news, the most widely cited being SPIKES, originally developed for use in oncology practice [28,29]. Although SPIKES and similar models have been recommended for adaptation to emergency practice [33,34], there has been little evaluation of their appropriateness or effectiveness in ED contexts. The majority of the evidence supporting these tools derives from settings where clinicians have more time to prepare, have more clinical information, can build deeper rapport or may have an existing relationship with the patient and relatives, and have access to dedicated private spaces; all of these are notably lacking for those working in emergency care. When evaluations of these frameworks are undertaken, they tend to prioritise clinician self-reported confidence and satisfaction, rather than patient or family perspectives.

To date, there has been no comprehensive review of the extent and nature of the literature specifically addressing the communication of bad news in the ED. Two current related reviews are addressing the breaking of bad news across all healthcare settings [35] and the communication of unexpected death [36]. Consequently, clinicians and educators lack a synthesis of what is known about effective practices, what guidance has been developed and implemented, and what evidence gaps remain in this area.

Given the diversity of communication practices, settings, professional roles, and cultural considerations across emergency care systems internationally, we chose a scoping review approach to address this problem. Scoping reviews are designed to comprehensively map the evidence in a field, regardless of study design or quality, with the aim of identifying key concepts, types of evidence, and gaps in the literature [37,38]. The present review was conducted to provide an accessible representation of available literature and inform the development of targeted research, training interventions, and policy initiatives to improve communication practices in the ED.

## Objectives

This scoping review aimed to map the extent, nature and characteristics of the literature on the delivery of bad news in adult emergency department contexts. In doing so, it sought to clarify what is known about how bad news is delivered in emergency care; the extent to which communication frameworks, tools and guidelines have been developed specifically for emergency practice or adapted from other areas of clinical care; and where important evidence gaps remain.

The review was guided by the following questions:

- What empirical, educational and clinician-facing literature has been published concerning the delivery of bad news in adult emergency department contexts, including studies, reviews, guidelines, editorials, commentaries, communication frameworks, tools and educational resources?
- What terminology is used to refer to conversations involving bad news within the literature related to emergency medicine practice?
- What is known about the experiences and views of healthcare staff of delivering bad news in emergency departments and those of patients and relatives receiving it?
- What barriers and facilitators influencing effective communication in the ED environment are reported?
- What outcomes associated with different approaches to delivering bad news are reported?
- What are the gaps in evidence that represent potential areas for further research, policy, and education?

In addressing these questions, the review seeks to support improvements in clinician training and patient-centred care for some of the most difficult conversations encountered in healthcare.

## Methods

### Protocol and registration

The protocol for this scoping review was developed in accordance with the methodology outlined by the Joanna Briggs Institute (JBI) for conducting scoping reviews [36] and is reported in accordance with the Preferred Reporting Items for Systematic Reviews and Meta-Analyses extension for Scoping Reviews (PRISMA-ScR). A completed PRISMA-ScR checklist is provided as Supplementary File 1. The protocol was registered prospectively on the Open Science Framework (OSF), https://osf.io/f6q59/overview.

### Eligibility criteria

The eligibility criteria were defined a priori using the Population-Concept-Context (PCC) framework recommended by the JBI. This approach is particularly suited to scoping reviews, which aim to map the breadth of evidence on a topic rather than answer narrowly focused clinical questions [38]. The criteria were designed to identify literature concerning the delivery of bad news in adult emergency department contexts, including empirical studies and clinician-facing publications such as reviews, guidelines, commentaries and educational articles. The inclusion and exclusion criteria are summarised in Table 1.

**Table 1:** Inclusion and exclusion criteria.

PCC element	Included	Excluded
**Population**	• Healthcare professionals involved in delivering emergency care, including doctors, nurses, allied health professionals and other clinicians working in, or contributing to care within, the emergency department. • Adult patients aged ≥18 years and relatives, friends or representatives receiving bad news in an emergency department context.	• Paediatric patients and their parents/caregivers; • studies focused only on children or young people; • studies involving healthcare professionals without relevance to emergency department care.
**Concept**	Communication involving the delivery of bad news, operationalised as communication relating to: • a terminal diagnosis where death is imminent or highly probable; • a serious, life-limiting or life-threatening diagnosis requiring admission, further investigation or treatment; • or the death of a patient being communicated to relatives or others.	• General communication-skills teaching or assessment not specifically related to bad news; • communication about routine care, discharge, consent or patient satisfaction where bad news was not a focus.
**Context**	• Physical emergency departments, accident and emergency departments, casualty departments, emergency rooms or equivalent international settings. • Simulation-based studies where the intervention or scenario was explicitly designed for emergency department clinicians or emergency department practice.	• Intensive care, inpatient wards, oncology, palliative care, primary care, outpatient or prehospital settings unless there was a clear emergency department focus. • Simulation studies not designed for emergency department practice
**Source types**	• Empirical studies; • reviews, guidelines, editorials, commentaries; • educational resources; • conference abstracts; and relevant grey literature.	• Sources without full text available in English; • sources where the full text could not be obtained; • duplicate publications unless they contained additional relevant data.

For the purposes of searching and screening, “bad news” was treated as a stable category, whilst recognising that the experience of news is subjective and will vary between patients, relatives and clinical situations. Paediatric emergency care was excluded because communication with children and caregivers involves distinct developmental, legal and relational considerations and is supported by a separate body of literature. Studies from non-ED settings were included only where the emergency department context was explicit. There is international variation in both how emergency departments function and how they are termed; the range of international terms was accounted for in the search strategy.

### Information sources

The search strategy aimed to comprehensively identify both published and grey literature. The following electronic databases were searched on 03/07/2025:

- MEDLINE (via Ovid)
- EMBASE (via Ovid)
- EMCARE
- CINAHL

Google Scholar was also search for journal literature (searched until 10 pages with no relevant papers identified)

Supplemental and grey literature sources were searched, including:

- The Anne Gray Library and Knowledge Services (LKS) Workshop repository
- the British Library;
- Social Science Research Network (SSRN);
- King’s Fund Library;
- TRIP database;
- Guidelines International;
- OpenAIRE and
- Virtual Health Library (VHL) Portal

Reference lists of all included publications and relevant reviews were also manually screened to identify further studies. The most recent search was executed prior to the commencement of title and abstract screening. Databases were selected in consultation with a clinical librarian to provide coverage of medicine, nursing, allied health literature relevant to emergency care communication. Grey literature sources were selected to identify guidelines, reports, educational materials and non-indexed clinician-facing publications relevant to emergency medicine practice.

### Search

The search strategy was designed to identify published, unpublished and grey literature sources and was structured to reflect: the population (healthcare providers and adult patients and relatives); the context (EDs and emergency care); and the concept (bad news delivery). Boolean operators were used and database specific index terms were used where available (e.g., Medical Subject Headings (MeSH) in Medline). Free text terms were searched in title/abstract fields. No date restrictions were applied. As EDs globally employ a wide range of professional groups, with different titles (doctor/physician, nurse, practitioner, etc.) adjacency terms were used to broaden the search. The search was adapted for each database. The full reproducible search strategies for all databases are included in Appendix I.

### Selection of sources

A three-step search process was followed:

1. An initial limited search of MEDLINE was performed to identify relevant keywords and index terms from titles, abstracts, and subject headings.
2. The identified terms were used to develop a comprehensive search strategy, which was adapted for each database.
3. The reference lists of included publications were hand-searched for additional studies in a snowballing technique.

All search results were exported to Rayyan [39] for deduplication. A data extraction form, adapted from JBI, was used. Two reviewers (GJ, SE) completed a pilot screening of 25 citations to ensure consistency and to test the data capture form.

The selection process followed a two-stage screening approach:

- Title and abstract screening: GJ and SE independently screened all titles and abstracts against the eligibility criteria, with disagreements resolved by discussion.
- **Full-text screening:** All potentially relevant studies were retrieved by GJ with checking by SE.

**Inclusion:**

- Adult participants (healthcare providers, patients or relatives)
- Bad news delivery (to include where death is imminent or likely, diagnosis of a serious illness and death notifications)
- ED setting

**Exclusion:**

- Paediatric patients
- General communication skills interventions, not specifically concerning bad news
- Not in an emergency setting

Reasons for exclusion of studies at the full-text stage were recorded. Discrepancies between reviewers were resolved by discussion. A third reviewer was available where discussion did not resolve disagreements, but this was not required.

The results of the selection process can be seen in the Preferred Reporting Items for Systematic Reviews and Meta-Analyses (PRISMA) flow diagram [38], Fig 1.

**Fig 1:**
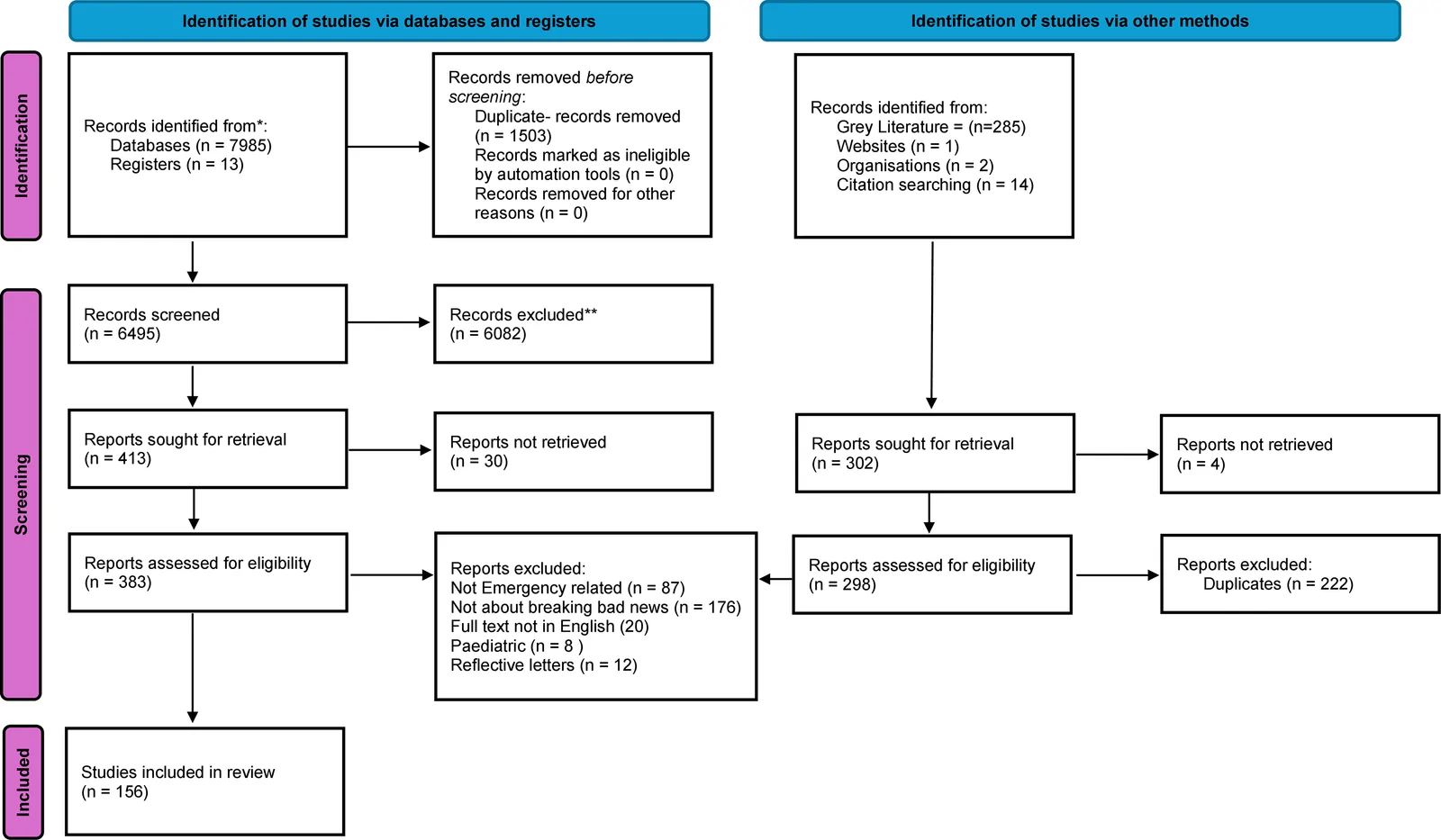
Prisma 2020 Flow diagram.

### Data extraction process

Data were extracted using a structured data extraction form adapted from the JBI scoping review template [38]. Some publications were international, with authorship from several countries. Where studies involved data collection the country of data collection was used as the study country. For editorial/review articles the country listed for the first author has been taken as the country of origin. There were no instances of data collection occurring in more than one country.

The adapted form added key components of the review, specifically and where applicable: Communication Framework used or referenced; Intervention; Barriers and Facilitators; primary outcome; terminology; key findings. The form was piloted on a sample of studies and refined iteratively to capture the relevant data fields comprehensively.

Data extraction was conducted independently by the lead author. SE extracted data for 10% of publications to ensure consistency. Any disagreements were resolved through discussion or adjudication by a third reviewer. If key data were missing or unclear, study authors were contacted for clarification where feasible.

### Data items

The following data items were extracted:

- **Publication Details:** Author(s), year of publication, country of origin, journal/source.
- **Study Characteristics:** Study design, aims, and methodology.
- **Participants:** Professional background of clinicians, characteristics of patients/relatives, sample size.
- **Context:** The characteristics of the ED setting (ED or simulated).
- **Bad News Delivery:** Described practices, protocols, frameworks, guidelines, or training interventions employed.
- **Terminology:** Terms used to describe and conceptualise communication practices.
- **Outcomes:** Patient and relative experiences, clinician experiences, patient and relative satisfaction, clinician confidence, and other relevant measures.
- **Barriers and Facilitators:** Factors that may influence the delivery of bad news in an ED setting (e.g., time, training, clinical scenarios).
- **Summary Findings:** Results and conclusions relevant to the review objectives.

### Critical appraisal of individual sources of evidence

In keeping with the scoping review methodology recommended by JBI and PRISMA-ScR, a critical appraisal of the methodological quality of included studies was not performed [38,40]. The objective of this review was to map the scope and nature of the evidence rather than to assess its quality.

### Synthesis of results

All data were synthesised narratively and tabulated to illustrate characteristics of included studies, the range of communication practices, and common barriers and facilitators. Very similar terminologies were collapsed where appropriate, such as *Breaking Bad News* and *Bad News* or *End-of-Life-Care* and *End of Life Care*. Reported findings were reviewed, charted, and grouped into broad categories that reflected recurrent concepts and patterns relevant to the review objectives, rather than re-analysing primary data.

## Results

Database searches identified 7,985 records and supplementary/grey literature searches identified a further 285 records. Following deduplication, title and abstract screening, and full-text assessment, 156 publications were included in the review (Fig 1). Reasons for exclusion at full-text stage included publications not being about communication in an emergency department, not being about bad news, and full text not being available in English. Full search strategies are provided in Appendix I. A complete source-level charting table for all included publications is provided in Appendix II. A complete source-level characteristics table for all included studies can be found at https://doi.org/10.17028/rd.lboro.33109310.v1

### Characteristics of included sources

This section provides a descriptive overview of the included publications, including country of origin, year of publication, source type, publication type and study design. Of the 156 included publications, 60 reported empirical data. Non-empirical sources included 51 reports of educational programs and 31 educational articles, with the remaining publications comprising other source types such as guidelines, letters, a book chapter, and a blog.

### Country of origin

Included publications originated from 26 countries across 6 continents, with Antarctica being the only continent not represented; a symbol map representation is available in Fig 2.

**Fig 2:**
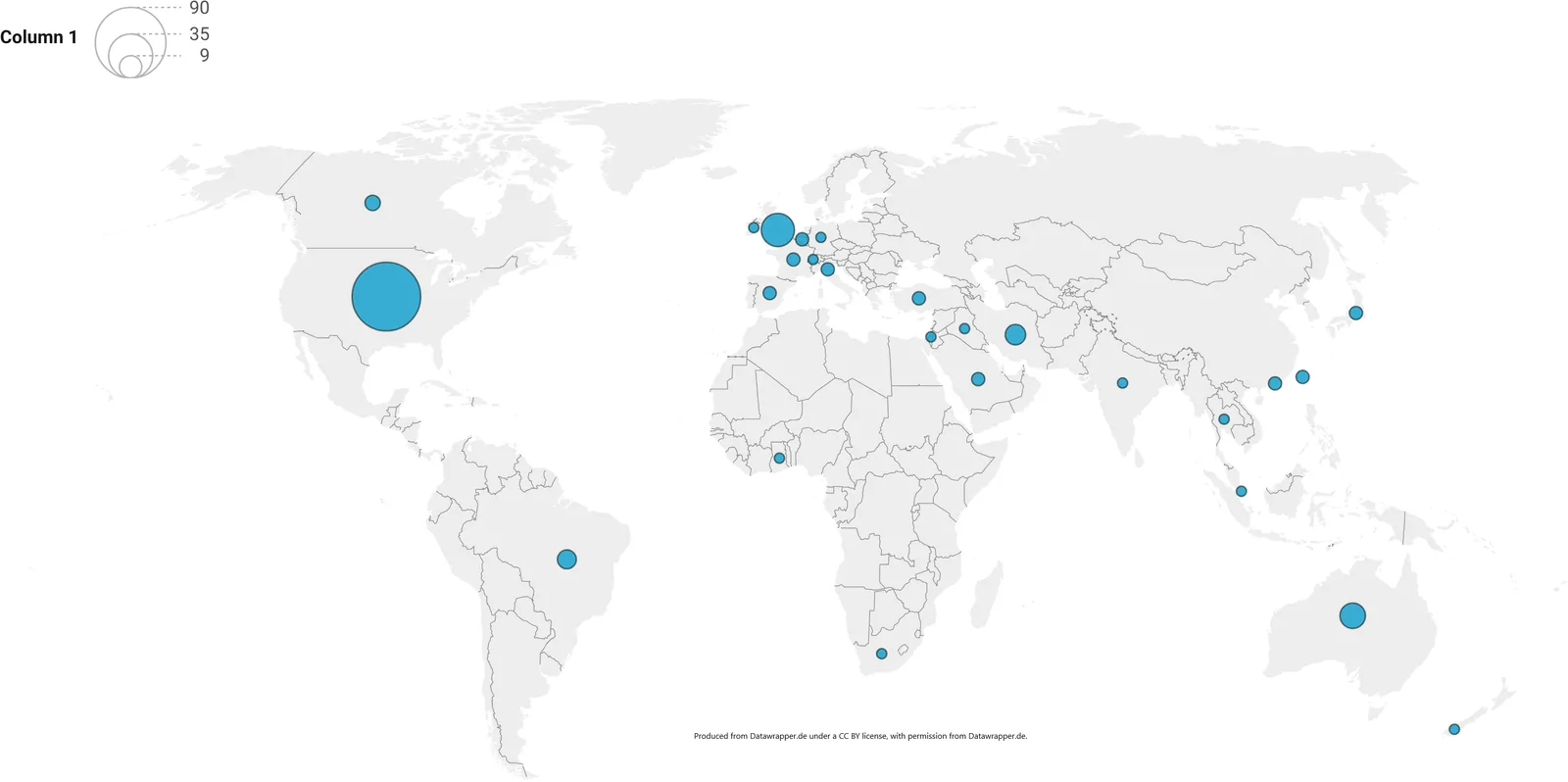
Country of origin.

The literature was dominated by publications from the United States (n = 85), followed by the United Kingdom (n = 18), Australia (n = 10), Iran (n = 6) and Brazil (n = 5). Most other countries contributed one or two publications. Overall, the evidence base was concentrated in North America and Europe, with smaller contributions from Asia, Oceania, South America and Africa.

Publication year trends (Fig 3) The earliest included publication was from the USA in 1979 [41] and the first non-US publication was from the UK in 1996 [42]. Publication numbers remained low over the following decades but increased from 2010 onwards. The highest number of publications was identified in 2023 (n = 17). Publications from 2025 were included for completeness, although the search captured only the first six months of that year.

**Fig 3:**
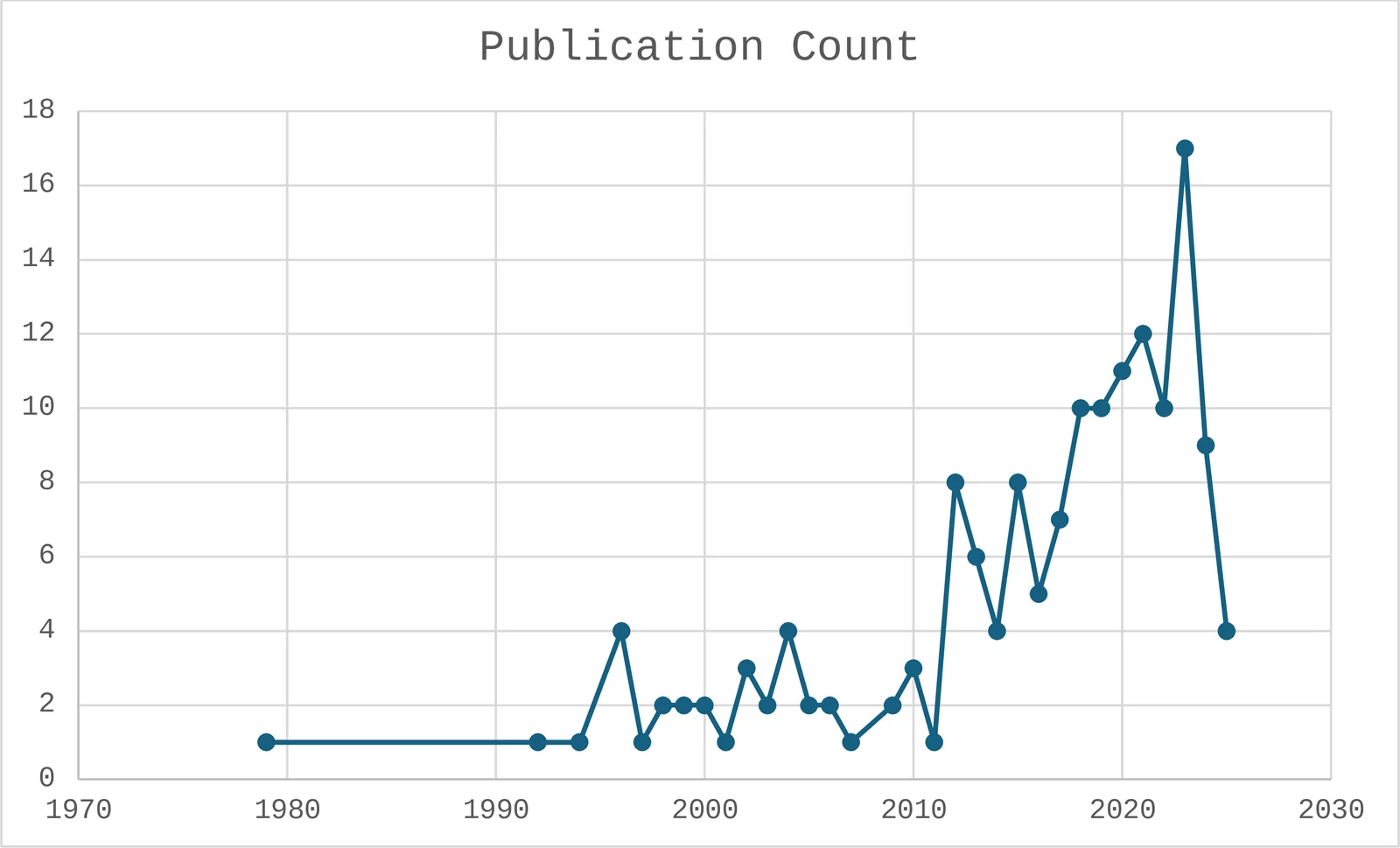
Publications over time.

### Sources

The majority of publications identified were from peer-reviewed journals (n = 122) with conference proceedings also forming a substantial part(n = 26). Peer-review status was unclear for a small number of publications, most commonly where journals were no longer in print, had mixed publication models, or provided educational content without a clearly stated review process. Three publications were classified as not peer reviewed: one commissioned article, one blog and one book chapter.

The majority of publications (n = 69) were in journals directly relevant to emergency medicine, followed by nursing (n = 24), palliative medicine (n = 21), general medicine (n = 10) and medical teaching and education journals (n = 9). Smaller numbers were published in social science, psychology/mental health, ethics/law and oncology journals, while 16 publications were difficult to classify by journal title.

Study and publication typesA range of study and publication types was identified, from empirical studies and training evaluations to non-research contributions such as editorials, commentaries, educational articles and guidelines (Table 2).

**Table 2:** Publication format and types.

Format	n papers	Percentage
Journal article	122	78
Abstract	29	19
Guideline	3	2
Book Chapter	1	0.6
Blog	1	0.6
**Total**	**156**	
**Type**		
**Empirical**	**60**	38
Survey	25	16
Interviews	15	10
Observational	6	4
Observational, simulation	6	4
Systematic Review	5	3
Other Review	2	1.3
Delphi	1	0.6
Research letter	1	0.6
**Non-empirical**	**96**	62
Report of educational program	51	33
Educational/Review	31	20
Editorial	4	3
Letter to the editor	3	2
Guideline	3	2
Book chapter	1	0.6
Blog	1	0.6
Personal reflection	1	0.6

Reports of educational programs formed the largest category, followed by educational articles. These publications were commonly intended to provide practical guidance for clinicians, often drawing on existing evidence or clinical experience.

Empirical studies most commonly used survey methods, followed by interview-based studies. Observational work was uncommon, and only a small number of studies examined real-world emergency department encounters directly through either direct observation or use of video recording. No experimental studies evaluating real-world emergency department bad news communication were identified. Source-level study and publication type classifications are provided in Appendix II.

### Domains

The search strategy was structured around three broad domains of bad news communication: terminal diagnosis, serious or life-limiting diagnosis, and death notification. Publications were distributed across all three domains, with the largest group addressing serious or life-limiting diagnoses (n = 71), followed by terminal diagnosis (n = 43) and death notification (n = 42) (Table 3).

**Table 3:** Domains.

Category / Subgroup	Count
**Serious/life-limiting diagnosis (requires admission/investigation/treatment)**	**71**
Giving News	61
Serious Illness Conversations	4
Early pregnancy loss/miscarriage	3
Needs of family of critically ill patients	3
**Terminal diagnosis (death imminent/highly probable)**	**43**
End-of-Life and Palliative Care	35
Goals of Care/Code Status	6
Critical Illness Discussion	2
**Death of a patient (communicated to relatives/friends)**	**42**

Within the serious or life-limiting diagnosis domain, most publications addressed the general activity of giving bad news in the emergency department, often in relation to training programmes or professional experience. Publications addressing terminal diagnosis most commonly focused on end-of-life or palliative care discussions, with fewer addressing goals of care, code status or critical illness discussions. Publications concerning the death of a patient included death notification practices, professional training and relatives’ experiences of receiving such news.

### Frameworks and tools

A range of communication frameworks and tools was identified across the included publications with 25 frameworks or tools identified across 80 publications. (Table 4). SPIKES [29] was the most frequently cited framework, followed by GRIEV_ING [30], NURSE [43], EMTalk [44], ABCDE [45], BREAKS [46] and VitalTalk [47]. Several publications, particularly editorials and educational articles, discussed more than one framework.

**Table 4:** Frameworks.

Framework/Tool	Mentions
SPIKES	37
GRIEV_ING	12
NURSE	9
EMTalk	6
ABCDE	5
BREAKS	4
VitalTalk	4
SHARE	3
Ask-Tell-Ask	2
Empathic Death Disclosure	2
Best Case/Worst Case	2
Kaye’s 10 Steps	2
8S	2
OUCHI	2
Natural Death	1
MTalk	1
ELNEC CRITICAL CARE MODULE	1
SILVER	1
I Wish vs I’m Sorry	1
PEWTER	1
Ask-Understand-Ask-Provide	1
ASKWIVES	1
SUNBURN	1
SPwICES	1
Calgary Cambridge Model	1
General principles and suggestions	6

Only three frameworks were developed specifically for emergency department practice: GRIEV_ING [30], 8S [48] and EMTalk [49]. GRIEV_ING focuses on death notification, while 8S was developed for breaking bad news by telephone during Covid-19. Most other frameworks originated in other clinical settings, particularly oncology, palliative care and critical care, and were advocated or adapted for use in emergency medicine. SPIKES [29] was the framework most frequently used in training programmes.

### Terminology used

A range of terms were employed across the publications to describe communication practices in ED contexts. Minor variants such as “breaking bad news” and “breaking the bad news” were standardised during data extraction to support comparison across sources.

“Breaking bad news” was the most frequently used (n=61), followed by “bad news” (n=24), “End-of-life” (n=25), “Death notification” (n=22), “Difficult conversations” (n=15) and “goals-of-care” (n=10). Less common terms were “serious illness” (n=6), “crisis/critical situations” (n=4), “death disclosure” (n=4), “news” (n=2) and “death” (n=2).

To examine conceptual overlap, related terms were grouped into broader categories: bad news, death, difficult interactions, end of life, goals of care, serious illness, compassion and news. The bad news category most frequently co-occurred with goals of care (n = 18), death-related communication (n = 15), end-of-life communication (n = 13) and difficult interactions (n = 12). This indicates that bad news was often used as an umbrella framing across several different forms of communication, rather than as a clearly bounded term.

Overall, the terminology was inconsistent. Death notification, end-of-life care, goals-of-care discussions, serious illness conversations and communicating early pregnancy loss were sometimes treated as distinct communication practices, but were also frequently subsumed within a broader bad news framing. This variation supports the need for clearer conceptual distinctions within future emergency department communication research.

### Stakeholder perspectives, experiences and needs

Studies examining stakeholder perspectives were unevenly distributed across healthcare staff, patients and relatives (Table 5).

**Table 5:** Stakeholder perspectives; study types.

Study design	Healthcare staff	Relatives/family	Patients
Interview	7	5	2
Other Qualitative	1	0	0
Review	0	2	1
Mixed methods	1	0	0
Survey	11	3	5
Total	20	10	8

Healthcare staff perspectives were most frequently represented (n = 20), with studies commonly addressing clinicians’ approaches to delivering bad news, perceived preparedness, barriers to communication, and the emotional and professional impact of these encounters. Reported metrics included self-reported confidence, comfort, perceived skills and identification of training or resource needs.

Patient and relative perspectives were less commonly represented. Patient-focused studies (n = 8) explored preferences for how difficult information was communicated, including timing, setting, directness, sensitivity, clarity and trust in clinicians. Studies involving relatives or family members (n = 10) addressed information needs, opportunities to ask questions, involvement in care planning or decision-making, and emotional support during or after communication.

Across stakeholder groups, patients and relatives most frequently emphasised honesty, clarity, clinician presence and emotional support. Clinicians more commonly highlighted constraints related to time, training, confidence and physical space. Overall, the literature gives greater attention to clinician perspectives than to the experiences and priorities of patients and relatives receiving bad news in emergency department contexts.

### Outcomes of training interventions

Of the 51 articles reporting an educational program, 36 reported an evaluation of a training intervention for delivering bad news in emergency department contexts. Most used self-report questionnaires, commonly before and after training or immediately after completion, to assess confidence, comfort, perceived competence or perceived usefulness. Likert-type scales were used in 30/36 studies. Thirteen evaluations were reported as conference proceedings or meeting reports, limiting the methodological detail available.

Reported outcomes were generally positive. Improvement in at least one assessed outcome was reported in 14/36 studies, while 6/36 specifically reported increased confidence or comfort following training. Confidence or comfort was the most common outcome domain overall (23/36), and was usually self-reported. Positive appraisals of training, such as perceived usefulness, acceptability or benefit, were reported in at least 15/36 studies. Fewer studies assessed skills, competence or protocol adherence.

Simulation-based interventions were the largest subgroup (23/36). Of these, 19 reported positive outcomes, two were neutral, and two did not report outcome data. Observer-rated assessments of competence were uncommon (5/36) and were all conducted in simulated or role-play settings. These assessments usually used locally developed checklists based on the communication framework being taught, with limited reporting of validation or inter-rater reliability. Two studies used the Breaking Bad News Assessment Scale and one used an adapted Kristjanson’s FAMCARE scale.

No studies assessed direct observation of real-world emergency department bad news conversations, and none reported patient- or relative-level outcomes. Overall, training interventions were generally reported as beneficial for clinicians, particularly in improving self-reported confidence and comfort, but whether these improvements translate into benefits for patients or relatives remains unknown.

#### Barriers.

Studies described consistent barriers to effective communication with patients and families in emergency department contexts. Time pressure was frequently reported, with clinicians describing the difficulty of balancing urgent clinical demands with sensitive communication. Lack of training and preparedness also contributed to reduced confidence when delivering bad news. Environmental factors, including interruptions, noise and lack of private space, further constrained communication. Barriers also arose from the clinical situation itself, particularly when deterioration was unexpected, prognosis was uncertain, or the patient was unstable. Together, these factors created circumstances in which timely, clear and compassionate communication was difficult to achieve.

#### Facilitators.

Studies also identified factors that supported effective communication in emergency department contexts. Preparation before the conversation, even when limited, helped clinicians structure discussions and gave patients or relatives time to process emerging information. The presence of additional staff could support both clinicians and families, particularly during emotionally demanding conversations. Access to a suitable private space was repeatedly described as important for sensitive communication. Some studies also reported that family presence during critical events, such as resuscitation, could provide transparency and reassurance. Overall, the facilitators identified across the literature emphasised preparation, privacy, staff support and opportunities for families to receive information and ask questions.

### Special circumstances – telephone conversations, COVID-19 and Artificial Intelligence

Several publications addressed circumstances requiring modification of usual approaches to delivering bad news. Eight publications discussed breaking bad news by telephone [48,50–57]. Although the earliest was published in 1999 and another in 2012, most were published from 2021 onwards, reflecting increased attention to remote communication during and after the COVID-19 pandemic.

Eight publications addressed breaking bad news during the COVID-19 pandemic [48,53,54,57–61]. Five of these focused specifically on telephone communication, while one described virtually mediated communication [61]. These publications highlighted the need for clinicians to adapt communication practices when family presence, face-to-face discussion and usual sources of support were restricted.

One publication examined the use of artificial intelligence, specifically ChatGPT, as an educational tool for breaking bad news [62]. This involved a single clinician engaging in a simulated conversation with ChatGPT in the role of the patient.

### Evidence gap

Despite the breadth of literature identified, empirical research examining real-world delivery of bad news in emergency departments was limited. Most studies focused on training interventions, simulation or clinician-reported experiences, with comparatively little direct observation of clinical practice. Only a small number of studies examined real emergency department encounters, including one using conversation analysis and two using ethnographic methods.

Patient and relative perspectives were also under-represented, and no studies evaluated the impact of specific communication practices on patient- or relative-level outcomes. The literature therefore provides limited evidence about how bad news conversations unfold in practice, which communication approaches are most helpful to recipients, or whether clinician-focused training translates into better experiences for patients and families.

Overall, the review identified a need for more empirical research in real-world emergency department settings, including interactional studies of naturally occurring conversations, studies foregrounding patient and relative perspectives, and evaluations using outcomes beyond clinician confidence or satisfaction.

## Discussion

This review maps a varied and uneven evidence base on how bad news is communicated in emergency departments (EDs), highlighting that ED communication training relies on frameworks from other specialities, despite little evidence of their effectiveness in real-world ED practice. The literature demonstrates the sustained professional concern about these conversations, demonstrated by the prominence of editorials, commentaries and educational articles seeking to inform and equip clinicians to fill the gap left by the lack of widespread formal guidance. However, much of the evidence is derived from training evaluations, simulation studies and clinician self-report, rather than direct observation of real-world practice; this means that practice and training may be shaped by expert opinion and extrapolated guidance, rather than evidence of what works in the ED. Across the included sources, “bad news” was used as an umbrella term encompassing death notification, end-of-life and goals-of-care discussions, serious illness conversations and other high-stakes encounters. This breadth is useful for mapping the field, but also highlights the lack of conceptual precision and emergency department-specific empirical grounding in the literature.

The distribution of publications should be interpreted in light of the review’s restriction to full-text English-language sources. The dominance of publications from the United States and United Kingdom may also reflect the organisation of emergency medicine as a distinct specialty in these settings, where specialty-specific training and research infrastructure may make communication research easier to develop. Publication numbers increased from 2010 onwards, suggesting growing recognition of bad news communication as part of emergency care, particularly in relation to ageing population in higher-income countries [63–65], end-of-life care and, more recently, remote communication during the COVID-19 pandemic. However, the concentration of evidence in particular healthcare systems limits the transferability of findings to settings where emergency care is organised differently or delivered by clinicians from multiple specialties.

The dependence on frameworks imported from other clinical domains, particularly oncology and palliative care, is notable. SPIKES [29] originally developed for oncology practice, was the most frequently cited framework and the most commonly used in training interventions. However, no studies were identified that established whether these frameworks are adopted in routine ED practice or whether their use improves outcomes for patients or relatives in this setting.

Frameworks developed for planned, private consultations may not transfer straightforwardly to the emergency department, where conversations often occur with limited preparation, incomplete information, lack of privacy, interruptions, and no prior clinician-patient relationship. The assumption that practices can be transferred from other clinical contexts warrants scrutiny as similar assumptions in other aspects of medical practice have previously been shown to be flawed [66–68]. The few ED-specific frameworks (GRIEV_ING [30], 8S [48] and the EMTalk training program [44]) represent an important recognition of this contextual specificity, but remain limited in number and scope. Future work should examine whether existing frameworks reflect the practical and interactional realities of emergency care, and whether ED-specific approaches better support clinicians, patients and relatives.

The terminology used across the literature highlights a further conceptual difficulty. “Bad news” was often used as a practical umbrella term, encompassing death notification, end-of-life and goals-of-care discussions, serious illness conversations and early pregnancy loss. This functional breadth was useful for structuring the review, but it risks obscuring important differences between communicative events. The disclosure of a poor prognosis, confirmation of a sudden death, discussion of treatment limitation and communication of early pregnancy loss are not necessarily experienced and managed in the same way, and may require different communicative approaches. Training courses and evaluative studies also commonly treat bad news as an umbrella category with specific scenarios nested within it [54,69].

This variability reflects a broader definitional problem. A common definition broadly describes bad news as information that negatively alters an individual’s expectations of the future [29,70]. Clinicians may categorise certain clinical situations as “bad news” while patients or relatives may experience the same information differently depending on prior expectations, uncertainty, relationships and context. For example, a serious diagnosis may bring relief if it resolves prolonged uncertainty, whereas apparently less serious information, such as restricted visiting, may be experienced as highly significant by patients or families [71]. Future research should therefore distinguish more clearly between different types of bad news encounter and examine what patients and relatives themselves consider to be bad news in emergency department contexts.

The imbalance in stakeholder perspectives is important because it shapes what the literature treats as evidence of successful communication. Although patient and relative perspectives were represented, the evidence base remains more strongly oriented towards clinician views and clinician-centred outcomes. Patients and relatives emphasised honesty, clarity, clinician presence and emotional support, whereas clinicians more often foregrounded time pressure, training needs, confidence and lack of private space. This imbalance is understandable, given the practical and ethical difficulties of involving patients and relatives during or after distressing ED encounters. A small number of observational studies involving patients have been undertaken, but consent processes, clinical acuity, bereavement and the appropriateness of approaching patients after receiving bad news all create challenges. Nonetheless, future research should treat patient and relative perspectives as central rather than supplementary, particularly if communication frameworks and training interventions are to reflect what matters most to those receiving bad news.

The barriers and facilitators identified across the literature indicate that communication in the ED is shaped by organisational conditions as well as individual clinician skill. Time pressure, crowding, interruptions, noise, lack of privacy and insufficient training were recurring barriers, while preparation, staff support and access to suitable private space were described as facilitators. The consistency of these findings suggests that difficulties in delivering bad news are not simply the result of inadequate communication skills, but are also produced by the structure and priorities of emergency care. Training may improve clinician confidence, but it cannot compensate for environments in which clinicians lack time, privacy or support for sensitive conversations.

There is also an intrinsic interactional difficulty in delivering bad news that was seldom addressed in the included literature. Maynard has described how bad news communication runs counter to the “benign order of everyday life”, in which interactions ordinarily maintain optimism and a positive trajectory [72]. In emergency care, clinicians may be required to disrupt this trajectory abruptly, often without prior relationship, full diagnostic certainty or sufficient time to prepare the recipient. This may help explain why delivering bad news is experienced as emotionally demanding and has been associated with stress and burn-out among emergency physicians [73].

The outcomes reported across training interventions were overwhelmingly positive, but most were clinician-centred and measured through self-report. Increased clinician confidence and comfort are important, particularly given the association between difficult communication, stress and burn-out in emergency clinicians [73–75]. However, confidence is not the same as communicative effectiveness, and tells us little about whether patients and relatives felt informed and supported, or longer-term psychosocial outcomes were improved. The scarcity of negative or neutral findings should also be interpreted cautiously, as training evaluations with unfavourable results may be less likely to be reported or published, and positive findings may partly reflect the choice of short-term self-reported outcomes. Poorly delivered bad news has been associated with patient complaints, distress, prolonged grief reactions and post-traumatic stress disorder [10,76,77], yet the impact of training or communication frameworks on these outcomes has not been assessed in ED contexts. Some studies assessed whether clinicians completed steps of a communication framework [61,69]. But checklist completion may not capture the relational, emotional or interactional quality of the conversation. Future evaluations should therefore move beyond clinician self-confidence and include patient- and relative-centred outcomes, observed communication practices and longer-term measures of impact.

These findings identify several priorities for future research. First, there is a need for empirical studies of naturally occurring emergency department conversations, including interactional studies that examine how bad news is delivered and received in real time. Second, future work should develop and evaluate ED-specific frameworks, informed by real-life observation and the experiences of clinicians, patients and relatives, rather than relying primarily on models developed in other clinical settings. Third, evaluations of training and communication frameworks should include outcomes that capture patient and relative perspectives, longer-term psychosocial effects, and observed communication practices, rather than relying exclusively on clinician confidence or satisfaction.

Future research should also address the organisational conditions that shape communication in emergency care. Space, staffing, crowding and organisational culture are already recognised challenges for emergency medicine more broadly [78–80], but their specific impact on bad news communication remains under-examined. Finally, the growth of telephone, virtual and artificial intelligence-mediated communication creates new areas for study. The COVID-19 pandemic showed how rapidly communication practices can change under systemic pressure; research should therefore move from a reactive stance to a more anticipatory one, examining how mediated forms of communication should be structured, supported and evaluated before they become embedded in routine practice.

### Strengths and limitations of this review

This scoping review provides a broad overview of international literature on the delivery of bad news in emergency department contexts. Its strengths include the use of a prospectively registered protocol, a structured PCC framework, a broad search strategy, inclusion of multiple source types, and source-level data charting. This allowed the review to map empirical studies, training evaluations, frameworks, guidelines, commentaries and other clinician-facing publications, and to identify conceptual and empirical gaps across the field. The inclusion of multiple source types was both a strength and a limitation. It allowed the review to map not only empirical studies, but also training evaluations, frameworks, guidelines, commentaries, conference abstracts and other clinician-facing publications. However, some included sources were not peer reviewed or provided limited methodological detail. These sources were useful for understanding how bad news communication is framed and discussed within emergency medicine, but they limit the strength of conclusions that can be drawn about effectiveness or impact.

Several limitations should be acknowledged. The review was restricted to full-text sources available in English, which may have introduced language bias and excluded relevant literature published in other languages. Translation using artificial intelligence tools was considered but not used because of concerns about accuracy [81,82]. The dominance of publications from the United States and United Kingdom may limit transferability to emergency care systems organised differently. The evidence base itself was also limited, with relatively few studies involving direct observation of real-world emergency department communication and limited representation of patient and relative perspectives. Finally, paediatric emergency medicine literature was excluded; this was intentional because communication with children and caregivers involves distinct developmental, legal and relational considerations, but it narrows the scope of the review.

### Implications for research, practice and policy

Future research should prioritise direct empirical study of naturally occurring bad news conversations in emergency department settings, including interactional studies and work foregrounding patient and relative perspectives. Training evaluations should move beyond clinician confidence and satisfaction, incorporating observed communication practices, patient- and relative-centred outcomes, and longer-term psychosocial measures. For practice, the findings support the development and evaluation of emergency department-specific frameworks that reflect the time pressure, uncertainty, interruptions and limited privacy of emergency care. At policy and service level, improving bad news communication should not be treated solely as an individual clinician training issue; it also requires attention to staffing numbers, providing space and privacy, senior support, and systems that allow sensitive conversations to occur safely and compassionately.

### Reflection

The lead author is a UK emergency physician, which informed the clinical interpretation of the findings but may also have shaped judgements about the relevance of sources to emergency department practice. To mitigate this, eligibility criteria and data-charting processes were agreed within a multidisciplinary research team.

## Conclusion

This scoping review identified a growing but uneven literature on the delivery of bad news in emergency department contexts. The evidence base is dominated by clinician-facing publications, training evaluations and frameworks adapted from other specialties, with limited direct observation of real-world emergency department practice and relatively few patient or relative perspectives. Existing training interventions are generally reported positively, but outcomes are usually clinician-centred and short term.

Future work should develop and evaluate emergency department-specific approaches to bad news communication, informed by real-world interactional evidence and by the priorities of patients, relatives and clinicians. Improving these conversations will require not only better training, but also organisational and policy attention to the conditions in which communication takes place, including privacy, staffing, time and support for clinicians and families.

## Supporting information

S1 File Appendix I full search strategy. (DOCX)

S2 File Appendix II included studies. (XLSX)

S3 File Supplement 1 PRSIMA ScR-checklist. (DOCX)
