# Supplementary material for: Understanding delivery of bad news in the emergency department: A scoping review
Source: PLoS One. 2026 Sep 28;21(9):e0358343. doi: 10.1371/journal.pone.0358343 (PMC13618904; doi:10.1371/journal.pone.0358343)
Supplement: Supplementary file 1 [file pone.0358343.s001.docx]

## Appendix I:

Electronic Databases

- MEDLINE (via OVID) (from January 1960 until the search date).
- EMBASE (via OVID) (from January 1960 until the search date).
- EMCARE
- CINAHL
- The Cochrane Central Register of Controlled Trials (CENTRAL) (The Cochrane Library, current issue).
- Google Scholar – 10 pages from the last relevant result will be reviewed.

**Other Useful Resources**

Several databases will be searched for relevant studies, reviews and guidelines (the reference lists of which will be hand-searched), including but not limited to: Cochrane Database of Systematic Reviews (CDSR); Health Technology Assessment Database (HTA Database); PROSPERO (International prospective register of systematic reviews) (from January 1960 until the search date).

**Unpublished/grey Literature (Electronic)**

Grey literature databases such as HMIC (Health Management Information Consortium), and Anne Gray LKS Workshop.

**Hand Searching**

Reference lists were followed up, scanning page by page.

**Medline**

1.	Emergency Medicine.mp. [mp=title, book title, abstract, original title, name of substance word, subject heading word, floating sub-heading word, keyword heading word, organism supplementary concept word, protocol supplementary concept word, rare disease supplementary concept word, unique identifier, synonyms, population supplementary concept word, anatomy supplementary concept word]

2.	ED.ti.

3.	Emergency Department.mp. [mp=title, book title, abstract, original title, name of substance word, subject heading word, floating sub-heading word, keyword heading word, organism supplementary concept word, protocol supplementary concept word, rare disease supplementary concept word, unique identifier, synonyms, population supplementary concept word, anatomy supplementary concept word]

4.	"A&E".ti.

5.	Casualty.mp. [mp=title, book title, abstract, original title, name of substance word, subject heading word, floating sub-heading word, keyword heading word, organism supplementary concept word, protocol supplementary concept word, rare disease supplementary concept word, unique identifier, synonyms, population supplementary concept word, anatomy supplementary concept word]

6.	Emergency Room.mp. [mp=title, book title, abstract, original title, name of substance word, subject heading word, floating sub-heading word, keyword heading word, organism supplementary concept word, protocol supplementary concept word, rare disease supplementary concept word, unique identifier, synonyms, population supplementary concept word, anatomy supplementary concept word]

7.	ER.ti.

8.	(Emergenc* adj3 (practitioner* or nurs* or clinician)).mp. [mp=title, book title, abstract, original title, name of substance word, subject heading word, floating sub-heading word, keyword heading word, organism supplementary concept word, protocol supplementary concept word, rare disease supplementary concept word, unique identifier, synonyms, population supplementary concept word, anatomy supplementary concept word]

9.	(Emergenc* adj3 (doctor* or physician* or specialist)).mp. [mp=title, book title, abstract, original title, name of substance word, subject heading word, floating sub-heading word, keyword heading word, organism supplementary concept word, protocol supplementary concept word, rare disease supplementary concept word, unique identifier, synonyms, population supplementary concept word, anatomy supplementary concept word]

10.	"Accident and Emergency".mp. [mp=title, book title, abstract, original title, name of substance word, subject heading word, floating sub-heading word, keyword heading word, organism supplementary concept word, protocol supplementary concept word, rare disease supplementary concept word, unique identifier, synonyms, population supplementary concept word, anatomy supplementary concept word]

11.	exp Emergency Service, Hospital/

12.	exp Emergency Medicine/

13.	1 or 2 or 3 or 4 or 5 or 6 or 7 or 8 or 9 or 10 or 11 or 12

14.	Difficult conversation*.mp. [mp=title, book title, abstract, original title, name of substance word, subject heading word, floating sub-heading word, keyword heading word, organism supplementary concept word, protocol supplementary concept word, rare disease supplementary concept word, unique identifier, synonyms, population supplementary concept word, anatomy supplementary concept word]

15.	Break* bad news.mp. [mp=title, book title, abstract, original title, name of substance word, subject heading word, floating sub-heading word, keyword heading word, organism supplementary concept word, protocol supplementary concept word, rare disease supplementary concept word, unique identifier, synonyms, population supplementary concept word, anatomy supplementary concept word]

16.	News delivery.mp. [mp=title, book title, abstract, original title, name of substance word, subject heading word, floating sub-heading word, keyword heading word, organism supplementary concept word, protocol supplementary concept word, rare disease supplementary concept word, unique identifier, synonyms, population supplementary concept word, anatomy supplementary concept word]

17.	Disclosure.mp. [mp=title, book title, abstract, original title, name of substance word, subject heading word, floating sub-heading word, keyword heading word, organism supplementary concept word, protocol supplementary concept word, rare disease supplementary concept word, unique identifier, synonyms, population supplementary concept word, anatomy supplementary concept word]

18.	Truth Disclosure/

19.	14 or 15 or 16 or 17 or 18

20.	Death.mp. [mp=title, book title, abstract, original title, name of substance word, subject heading word, floating sub-heading word, keyword heading word, organism supplementary concept word, protocol supplementary concept word, rare disease supplementary concept word, unique identifier, synonyms, population supplementary concept word, anatomy supplementary concept word]

21.	Dying.mp. [mp=title, book title, abstract, original title, name of substance word, subject heading word, floating sub-heading word, keyword heading word, organism supplementary concept word, protocol supplementary concept word, rare disease supplementary concept word, unique identifier, synonyms, population supplementary concept word, anatomy supplementary concept word]

22.	Died.mp. [mp=title, book title, abstract, original title, name of substance word, subject heading word, floating sub-heading word, keyword heading word, organism supplementary concept word, protocol supplementary concept word, rare disease supplementary concept word, unique identifier, synonyms, population supplementary concept word, anatomy supplementary concept word]

23.	Terminal Care.mp. [mp=title, book title, abstract, original title, name of substance word, subject heading word, floating sub-heading word, keyword heading word, organism supplementary concept word, protocol supplementary concept word, rare disease supplementary concept word, unique identifier, synonyms, population supplementary concept word, anatomy supplementary concept word]

24.	Terminal illness.mp. [mp=title, book title, abstract, original title, name of substance word, subject heading word, floating sub-heading word, keyword heading word, organism supplementary concept word, protocol supplementary concept word, rare disease supplementary concept word, unique identifier, synonyms, population supplementary concept word, anatomy supplementary concept word]

25.	Palliative.mp. [mp=title, book title, abstract, original title, name of substance word, subject heading word, floating sub-heading word, keyword heading word, organism supplementary concept word, protocol supplementary concept word, rare disease supplementary concept word, unique identifier, synonyms, population supplementary concept word, anatomy supplementary concept word]

26.	End of life.mp. [mp=title, book title, abstract, original title, name of substance word, subject heading word, floating sub-heading word, keyword heading word, organism supplementary concept word, protocol supplementary concept word, rare disease supplementary concept word, unique identifier, synonyms, population supplementary concept word, anatomy supplementary concept word]

27.	Attitude to Death/

28.	death/ or exp death, sudden/ or drowning/ or infant death/ or sudden infant death/

29.	terminal care/ or hospice care/ or resuscitation orders/

30.	Palliative Care/

31.	20 or 21 or 22 or 23 or 24 or 25 or 26 or 27 or 28 or 29 or 30

32.	"Communicat*".m_titl.

33.	"Consult*".m_titl.

34.	Communication/

35.	nurse-patient relations/ or physician-patient relations/

36.	32 or 33 or 34 or 35

37.	31 and 36

38.	19 or 37

39.	13 and 38

**EMBASE**

1.	Emergency Medicine.mp. [mp=title, book title, abstract, original title, name of substance word, subject heading word, floating sub-heading word, keyword heading word, organism supplementary concept word, protocol supplementary concept word, rare disease supplementary concept word, unique identifier, synonyms, population supplementary concept word, anatomy supplementary concept word]

2.	ED.ti.

3.	Emergency Department.mp. [mp=title, book title, abstract, original title, name of substance word, subject heading word, floating sub-heading word, keyword heading word, organism supplementary concept word, protocol supplementary concept word, rare disease supplementary concept word, unique identifier, synonyms, population supplementary concept word, anatomy supplementary concept word]

4.	"A&E".ti.

5.	Casualty.mp. [mp=title, book title, abstract, original title, name of substance word, subject heading word, floating sub-heading word, keyword heading word, organism supplementary concept word, protocol supplementary concept word, rare disease supplementary concept word, unique identifier, synonyms, population supplementary concept word, anatomy supplementary concept word]

6.	Emergency Room.mp. [mp=title, book title, abstract, original title, name of substance word, subject heading word, floating sub-heading word, keyword heading word, organism supplementary concept word, protocol supplementary concept word, rare disease supplementary concept word, unique identifier, synonyms, population supplementary concept word, anatomy supplementary concept word]

7.	ER.ti.

8.	(Emergenc* adj3 (practitioner* or nurs* or clinician)).mp. [mp=title, book title, abstract, original title, name of substance word, subject heading word, floating sub-heading word, keyword heading word, organism supplementary concept word, protocol supplementary concept word, rare disease supplementary concept word, unique identifier, synonyms, population supplementary concept word, anatomy supplementary concept word]

9.	(Emergenc* adj3 (doctor* or physician* or specialist)).mp. [mp=title, book title, abstract, original title, name of substance word, subject heading word, floating sub-heading word, keyword heading word, organism supplementary concept word, protocol supplementary concept word, rare disease supplementary concept word, unique identifier, synonyms, population supplementary concept word, anatomy supplementary concept word]

10.	"Accident and Emergency".mp. [mp=title, book title, abstract, original title, name of substance word, subject heading word, floating sub-heading word, keyword heading word, organism supplementary concept word, protocol supplementary concept word, rare disease supplementary concept word, unique identifier, synonyms, population supplementary concept word, anatomy supplementary concept word]

11.	emergency health service/ or emergency ward/ or hospital emergency service/

12.	emergency medicine/ or pediatric emergency medicine/ or emergency nursing/

13.	1 or 2 or 3 or 4 or 5 or 6 or 7 or 8 or 9 or 10 or 11 or 12

14.	Difficult conversation*.mp. [mp=title, book title, abstract, original title, name of substance word, subject heading word, floating sub-heading word, keyword heading word, organism supplementary concept word, protocol supplementary concept word, rare disease supplementary concept word, unique identifier, synonyms, population supplementary concept word, anatomy supplementary concept word]

15.	Break* bad news.mp. [mp=title, book title, abstract, original title, name of substance word, subject heading word, floating sub-heading word, keyword heading word, organism supplementary concept word, protocol supplementary concept word, rare disease supplementary concept word, unique identifier, synonyms, population supplementary concept word, anatomy supplementary concept word]

16.	News delivery.mp. [mp=title, book title, abstract, original title, name of substance word, subject heading word, floating sub-heading word, keyword heading word, organism supplementary concept word, protocol supplementary concept word, rare disease supplementary concept word, unique identifier, synonyms, population supplementary concept word, anatomy supplementary concept word]

17.	Disclosure.mp. [mp=title, book title, abstract, original title, name of substance word, subject heading word, floating sub-heading word, keyword heading word, organism supplementary concept word, protocol supplementary concept word, rare disease supplementary concept word, unique identifier, synonyms, population supplementary concept word, anatomy supplementary concept word]

18.	Truth Disclosure/

19.	14 or 15 or 16 or 17 or 18

20.	Death.mp. [mp=title, book title, abstract, original title, name of substance word, subject heading word, floating sub-heading word, keyword heading word, organism supplementary concept word, protocol supplementary concept word, rare disease supplementary concept word, unique identifier, synonyms, population supplementary concept word, anatomy supplementary concept word]

21.	Dying.mp. [mp=title, book title, abstract, original title, name of substance word, subject heading word, floating sub-heading word, keyword heading word, organism supplementary concept word, protocol supplementary concept word, rare disease supplementary concept word, unique identifier, synonyms, population supplementary concept word, anatomy supplementary concept word]

22.	Died.mp. [mp=title, book title, abstract, original title, name of substance word, subject heading word, floating sub-heading word, keyword heading word, organism supplementary concept word, protocol supplementary concept word, rare disease supplementary concept word, unique identifier, synonyms, population supplementary concept word, anatomy supplementary concept word]

23.	Terminal Care.mp. [mp=title, book title, abstract, original title, name of substance word, subject heading word, floating sub-heading word, keyword heading word, organism supplementary concept word, protocol supplementary concept word, rare disease supplementary concept word, unique identifier, synonyms, population supplementary concept word, anatomy supplementary concept word]

24.	Terminal illness.mp. [mp=title, book title, abstract, original title, name of substance word, subject heading word, floating sub-heading word, keyword heading word, organism supplementary concept word, protocol supplementary concept word, rare disease supplementary concept word, unique identifier, synonyms, population supplementary concept word, anatomy supplementary concept word]

25.	Palliative.mp. [mp=title, book title, abstract, original title, name of substance word, subject heading word, floating sub-heading word, keyword heading word, organism supplementary concept word, protocol supplementary concept word, rare disease supplementary concept word, unique identifier, synonyms, population supplementary concept word, anatomy supplementary concept word]

26.	End of life.mp. [mp=title, book title, abstract, original title, name of substance word, subject heading word, floating sub-heading word, keyword heading word, organism supplementary concept word, protocol supplementary concept word, rare disease supplementary concept word, unique identifier, synonyms, population supplementary concept word, anatomy supplementary concept word]

27.	Attitude to Death/

28.	death/ or exp death, sudden/ or drowning/ or infant death/ or sudden infant death/

29.	terminal care/ or hospice care/ or resuscitation orders/

30.	Palliative Care/

31.	20 or 21 or 22 or 23 or 24 or 25 or 26 or 27 or 28 or 29 or 30

32.	"Communicat*".m_titl.

33.	"Consult*".m_titl.

34.	*Communication/

35.	nurse-patient relations/ or physician-patient relations/

36.	32 or 33 or 34 or 35

37.	31 and 36

38.	19 or 37

39.	13 and 38

**EMCARE**

1.	Emergency Medicine.mp. [mp=title, book title, abstract, original title, name of substance word, subject heading word, floating sub-heading word, keyword heading word, organism supplementary concept word, protocol supplementary concept word, rare disease supplementary concept word, unique identifier, synonyms, population supplementary concept word, anatomy supplementary concept word]

2.	ED.ti.

3.	Emergency Department.mp. [mp=title, book title, abstract, original title, name of substance word, subject heading word, floating sub-heading word, keyword heading word, organism supplementary concept word, protocol supplementary concept word, rare disease supplementary concept word, unique identifier, synonyms, population supplementary concept word, anatomy supplementary concept word]

4.	"A&E".ti.

5.	Casualty.mp. [mp=title, book title, abstract, original title, name of substance word, subject heading word, floating sub-heading word, keyword heading word, organism supplementary concept word, protocol supplementary concept word, rare disease supplementary concept word, unique identifier, synonyms, population supplementary concept word, anatomy supplementary concept word]

6.	Emergency Room.mp. [mp=title, book title, abstract, original title, name of substance word, subject heading word, floating sub-heading word, keyword heading word, organism supplementary concept word, protocol supplementary concept word, rare disease supplementary concept word, unique identifier, synonyms, population supplementary concept word, anatomy supplementary concept word]

7.	ER.ti.

8.	(Emergenc* adj3 (practitioner* or nurs* or clinician)).mp. [mp=title, book title, abstract, original title, name of substance word, subject heading word, floating sub-heading word, keyword heading word, organism supplementary concept word, protocol supplementary concept word, rare disease supplementary concept word, unique identifier, synonyms, population supplementary concept word, anatomy supplementary concept word]

9.	(Emergenc* adj3 (doctor* or physician* or specialist)).mp. [mp=title, book title, abstract, original title, name of substance word, subject heading word, floating sub-heading word, keyword heading word, organism supplementary concept word, protocol supplementary concept word, rare disease supplementary concept word, unique identifier, synonyms, population supplementary concept word, anatomy supplementary concept word]

10.	"Accident and Emergency".mp. [mp=title, book title, abstract, original title, name of substance word, subject heading word, floating sub-heading word, keyword heading word, organism supplementary concept word, protocol supplementary concept word, rare disease supplementary concept word, unique identifier, synonyms, population supplementary concept word, anatomy supplementary concept word]

11.	emergency health service/ or emergency ward/ or hospital emergency service/

12.	emergency medicine/ or pediatric emergency medicine/ or emergency nursing/

13.	1 or 2 or 3 or 4 or 5 or 6 or 7 or 8 or 9 or 10 or 11 or 12

14.	Difficult conversation*.mp. [mp=title, book title, abstract, original title, name of substance word, subject heading word, floating sub-heading word, keyword heading word, organism supplementary concept word, protocol supplementary concept word, rare disease supplementary concept word, unique identifier, synonyms, population supplementary concept word, anatomy supplementary concept word]

15.	Break* bad news.mp. [mp=title, book title, abstract, original title, name of substance word, subject heading word, floating sub-heading word, keyword heading word, organism supplementary concept word, protocol supplementary concept word, rare disease supplementary concept word, unique identifier, synonyms, population supplementary concept word, anatomy supplementary concept word]

16.	News delivery.mp. [mp=title, book title, abstract, original title, name of substance word, subject heading word, floating sub-heading word, keyword heading word, organism supplementary concept word, protocol supplementary concept word, rare disease supplementary concept word, unique identifier, synonyms, population supplementary concept word, anatomy supplementary concept word]

17.	Disclosure.mp. [mp=title, book title, abstract, original title, name of substance word, subject heading word, floating sub-heading word, keyword heading word, organism supplementary concept word, protocol supplementary concept word, rare disease supplementary concept word, unique identifier, synonyms, population supplementary concept word, anatomy supplementary concept word]

18.	Truth Disclosure/

19.	14 or 15 or 16 or 17 or 18

20.	Death.mp. [mp=title, book title, abstract, original title, name of substance word, subject heading word, floating sub-heading word, keyword heading word, organism supplementary concept word, protocol supplementary concept word, rare disease supplementary concept word, unique identifier, synonyms, population supplementary concept word, anatomy supplementary concept word]

21.	Dying.mp. [mp=title, book title, abstract, original title, name of substance word, subject heading word, floating sub-heading word, keyword heading word, organism supplementary concept word, protocol supplementary concept word, rare disease supplementary concept word, unique identifier, synonyms, population supplementary concept word, anatomy supplementary concept word]

22.	Died.mp. [mp=title, book title, abstract, original title, name of substance word, subject heading word, floating sub-heading word, keyword heading word, organism supplementary concept word, protocol supplementary concept word, rare disease supplementary concept word, unique identifier, synonyms, population supplementary concept word, anatomy supplementary concept word]

23.	Terminal Care.mp. [mp=title, book title, abstract, original title, name of substance word, subject heading word, floating sub-heading word, keyword heading word, organism supplementary concept word, protocol supplementary concept word, rare disease supplementary concept word, unique identifier, synonyms, population supplementary concept word, anatomy supplementary concept word]

24.	Terminal illness.mp. [mp=title, book title, abstract, original title, name of substance word, subject heading word, floating sub-heading word, keyword heading word, organism supplementary concept word, protocol supplementary concept word, rare disease supplementary concept word, unique identifier, synonyms, population supplementary concept word, anatomy supplementary concept word]

25.	Palliative.mp. [mp=title, book title, abstract, original title, name of substance word, subject heading word, floating sub-heading word, keyword heading word, organism supplementary concept word, protocol supplementary concept word, rare disease supplementary concept word, unique identifier, synonyms, population supplementary concept word, anatomy supplementary concept word]

26.	End of life.mp. [mp=title, book title, abstract, original title, name of substance word, subject heading word, floating sub-heading word, keyword heading word, organism supplementary concept word, protocol supplementary concept word, rare disease supplementary concept word, unique identifier, synonyms, population supplementary concept word, anatomy supplementary concept word]

27.	Attitude to Death/

28.	death/ or exp death, sudden/ or drowning/ or infant death/ or sudden infant death/

29.	terminal care/ or hospice care/ or resuscitation orders/

30.	Palliative Care/

31.	20 or 21 or 22 or 23 or 24 or 25 or 26 or 27 or 28 or 29 or 30

32.	"Communicat*".m_titl.

33.	"Consult*".m_titl.

34.	*Communication/

35.	nurse-patient relations/ or physician-patient relations/

36.	32 or 33 or 34 or 35

37.	31 and 36

38.	19 or 37

39.	13 and 38

**CINAHL Ultimate**

S1	Emergency Medicine	Expanders - Apply equivalent subjects

Search modes - Proximity	Interface - EBSCOhost Research Databases

Search Screen - Advanced Search

S2	TI ED	Expanders - Apply equivalent subjects

Search modes - Proximity	Interface - EBSCOhost Research Databases

Search Screen - Advanced Search

S3	Emergency Department	Expanders - Apply equivalent subjects

Search modes - Proximity	Interface - EBSCOhost Research Databases

Search Screen - Advanced Search

S4	TI "A&E"	Expanders - Apply equivalent subjects

Search modes - Proximity	Interface - EBSCOhost Research Databases

Search Screen - Advanced Search

S5	Casualty	Expanders - Apply equivalent subjects

Search modes - Proximity	Interface - EBSCOhost Research Databases

Search Screen - Advanced Search

S6	Emergency Room	Expanders - Apply equivalent subjects

Search modes - Proximity	Interface - EBSCOhost Research Databases

Search Screen - Advanced Search

S7	TI ER	Expanders - Apply equivalent subjects

Search modes - Proximity	Interface - EBSCOhost Research Databases

Search Screen - Advanced Search

S8	(Emergenc* N3 (practitioner* or nurs* or clinician))	Expanders - Apply equivalent subjects

Search modes - Proximity	Interface - EBSCOhost Research Databases

Search Screen - Advanced Search

S9	(Emergenc* N3 (doctor* or physician* or specialist))	Expanders - Apply equivalent subjects

Search modes - Proximity	Interface - EBSCOhost Research Databases

Search Screen - Advanced Search

S10	"Accident and Emergency"	Expanders - Apply equivalent subjects

Search modes - Proximity	Interface - EBSCOhost Research Databases

Search Screen - Advanced Search

S11	(MH "Emergency Medical Services") OR (MH "Emergency Service") OR (MH "Trauma Centers")	Expanders - Apply equivalent subjects

Search modes - Proximity	Interface - EBSCOhost Research Databases

Search Screen - Advanced Search

S12	(MH "Emergency Nurses") OR (MH "Physicians, Emergency") OR (MH "Emergency Patients") OR (MH "Emergency Service+") OR (MH "Emergency Nursing+") OR (MH "Emergency Medicine") OR (MH "Emergency Care") OR (MH "Emergency Room Visits") OR (MH "Pediatric Emergency Nursing") OR (MH "Emergency Medical Services")	Expanders - Apply equivalent subjects

Search modes - Proximity	Interface - EBSCOhost Research Databases

Search Screen - Advanced Search

S13	S1 OR S2 OR S3 OR S4 OR S5 OR S6 OR S7 OR S8 OR S9 OR S10 OR S11 OR S12	Expanders - Apply equivalent subjects

Search modes - Proximity	Interface - EBSCOhost Research Databases

Search Screen - Advanced Search

S14	Difficult conversation*	Expanders - Apply equivalent subjects

Search modes - Proximity	Interface - EBSCOhost Research Databases

Search Screen - Advanced Search

S15	Break* bad news	Expanders - Apply equivalent subjects

Search modes - Proximity	Interface - EBSCOhost Research Databases

Search Screen - Advanced Search

S16	News delivery	Expanders - Apply equivalent subjects

Search modes - Proximity	Interface - EBSCOhost Research Databases

Search Screen - Advanced Search

S17	Disclosure	Expanders - Apply equivalent subjects

Search modes - Proximity	Interface - EBSCOhost Research Databases

Search Screen - Advanced Search

S18	Truth disclosure/	Expanders - Apply equivalent subjects

Search modes - Proximity	Interface - EBSCOhost Research Databases

Search Screen - Advanced Search

S19	S14 OR S15 OR S16 OR S17 OR S18	Expanders - Apply equivalent subjects

Search modes - Proximity	Interface - EBSCOhost Research Databases

Search Screen - Advanced Search

S20	Death	Expanders - Apply equivalent subjects

Search modes - Proximity	Interface - EBSCOhost Research Databases

Search Screen - Advanced Search

S21	Dying	Expanders - Apply equivalent subjects

Search modes - Proximity	Interface - EBSCOhost Research Databases

Search Screen - Advanced Search

S22	Died	Expanders - Apply equivalent subjects

Search modes - Proximity	Interface - EBSCOhost Research Databases

Search Screen - Advanced Search

S23	Terminal Care	Expanders - Apply equivalent subjects

Search modes - Proximity	Interface - EBSCOhost Research Databases

Search Screen - Advanced Search

S24	Terminal Illness	Expanders - Apply equivalent subjects

Search modes - Proximity	Interface - EBSCOhost Research Databases

Search Screen - Advanced Search

S25	Palliative	Expanders - Apply equivalent subjects

Search modes - Proximity	Interface - EBSCOhost Research Databases

Search Screen - Advanced Search

S26	End of life	Expanders - Apply equivalent subjects

Search modes - Proximity	Interface - EBSCOhost Research Databases

Search Screen - Advanced Search

S27	Attitude to Death	Expanders - Apply equivalent subjects

Search modes - Proximity	Interface - EBSCOhost Research Databases

Search Screen - Advanced Search

S28	Death OR death, sudden OR drowning OR infant death OR sudden infant death	Expanders - Apply equivalent subjects

Search modes - Proximity	Interface - EBSCOhost Research Databases

Search Screen - Advanced Search

S29	Terminal care OR hospice care OR resuscitation orders	Expanders - Apply equivalent subjects

Search modes - Proximity	Interface - EBSCOhost Research Databases

Search Screen - Advanced Search

S30	Palliative care	Expanders - Apply equivalent subjects

Search modes - Proximity	Interface - EBSCOhost Research Databases

Search Screen - Advanced Search

S31	S20 or S21 or S22 or S23 or S24 or S25 or S26 or S27 or S28 or S29 or S30	Expanders - Apply equivalent subjects

Search modes - Proximity	Interface - EBSCOhost Research Databases

Search Screen - Advanced Search

S32	Communicat*	Expanders - Apply equivalent subjects

Search modes - Proximity	Interface - EBSCOhost Research Databases

Search Screen - Advanced Search

S33	Consult*	Expanders - Apply equivalent subjects

Search modes - Proximity	Interface - EBSCOhost Research Databases

Search Screen - Advanced Search

S34	Nurse-patient relations OR physician-patient relations	Expanders - Apply equivalent subjects

Search modes - Proximity	Interface - EBSCOhost Research Databases

Search Screen - Advanced Search

S35	S32 OR S33 OR S34	Expanders - Apply equivalent subjects

Search modes - Proximity	Interface - EBSCOhost Research Databases

Search Screen - Advanced Search

S36	S31 AND S35	Expanders - Apply equivalent subjects

Search modes - Proximity	Interface - EBSCOhost Research Databases

Search Screen - Advanced Search

S37	S19 OR S36	Expanders - Apply equivalent subjects

Search modes - Proximity	Interface - EBSCOhost Research Databases

Search Screen - Advanced Search

S38	S13 and S37	Expanders - Apply equivalent subjects

Search modes - Proximity	Interface - EBSCOhost Research Databases

Search Screen - Advanced Search

Database - CINAHL Ultimate
